# Supplementary material for: An aberrant phase transition of stress granules triggered by misfolded protein and prevented by chaperone function
Source: EMBO J. 2017 Apr 4;36(12):1669–87. doi: 10.15252/embj.201695957 (PMC5470046; doi:10.15252/embj.201695957)
Supplement: Supplementary file 1 — Appendix [file EMBJ-36-1669-s001.pdf]

Appendix

Table of Contents

Appendix Figure S1. Characterization of purified Ubc9TS and Ubc9WT. 2

Appendix Figure S2. Colocalization of misfolded proteins and heat stress-induced SGs.......... 3

Appendix Figure S3. FRAP analysis of SOD1(A4V) in SGs.. 4

Appendix Figure S4. Colocalization analysis of SOD1(A4V) and RBPs inside SGs. 4

Appendix Figure S5. Properties of SGs enriched for Ubc9TS or SOD1(A4V). 5

Appendix Figure S6. Correlation between HSP70 and SOD1(A4V) enrichment in SGs. 6

Appendix Figure S7. Accumulation of DRiPs in SGs.. 6

References.. 7

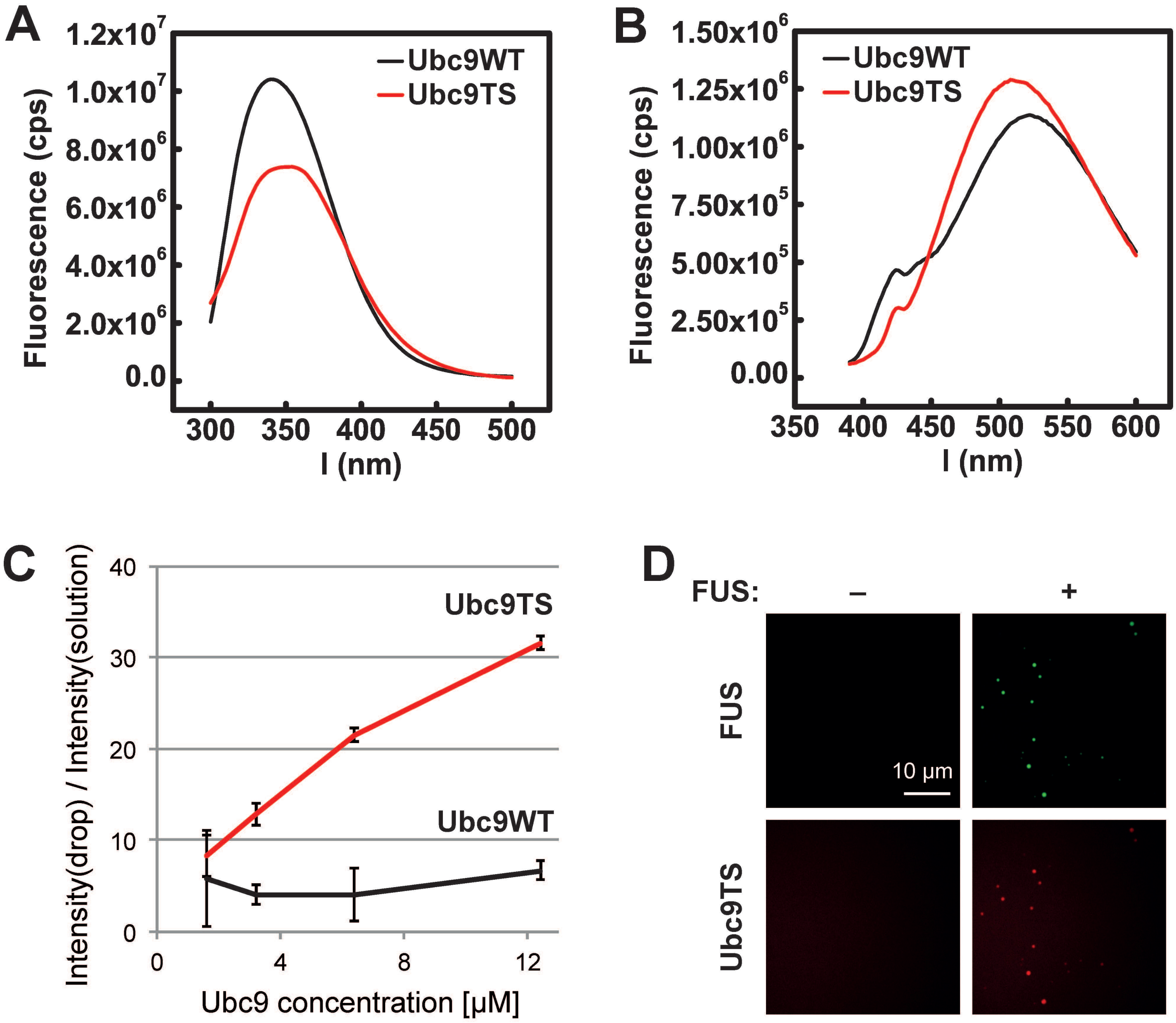

Appendix Figure S1. Characterization of purified Ubc9TS and Ubc9WT.

(A) Fluorescence spectra of 1 μM Ubc9WT and Ubc9TS. Excitation was at 275 nm. Fluorescence intensity is shown in cps (counts per second). As expected for an unfolded and less compact state (Lakowicz 2013), the fluorescence intensity of Ubc9TS is reduced and the intensity maximum is shifted from 340 nm to 355 nm compared to the control protein Ubc9WT.

(B) Fluorescence spectra of 100 μM ANS (1-anilinonaphthalene-8-sulfonic acid) incubated with 1 μM Ubc9WT or Ubc9TS. Excitation was at 370 nm. ANS exhibits strong fluorescence when bound to hydrophobic residues (Slavík 1982; Yan-Lin et al. 1998). Higher fluorescence of ANS suggests higher surface hydrophobicity.

(C) Quantification of Ubc9 accumulation in FUS droplets. Fluorescence intensities of Ubc9 in the FUS droplets and in the solution were measured at various concentrations of Ubc9WT or Ubc9TS. The fluorescence intensity ratios (Ubc9 signal inside the droplets / Ubc9 signal outside the droplets) were plotted. Error bars = SD.

(D) 4 μM Ubc9TS (1:10 mixture of Cy3-labeled:unlabeled) was imaged either alone (left) or in the presence of 5 μM FUS(G156E)-GFP (right). Ubc9TS remained in solution without the presence of FUS.

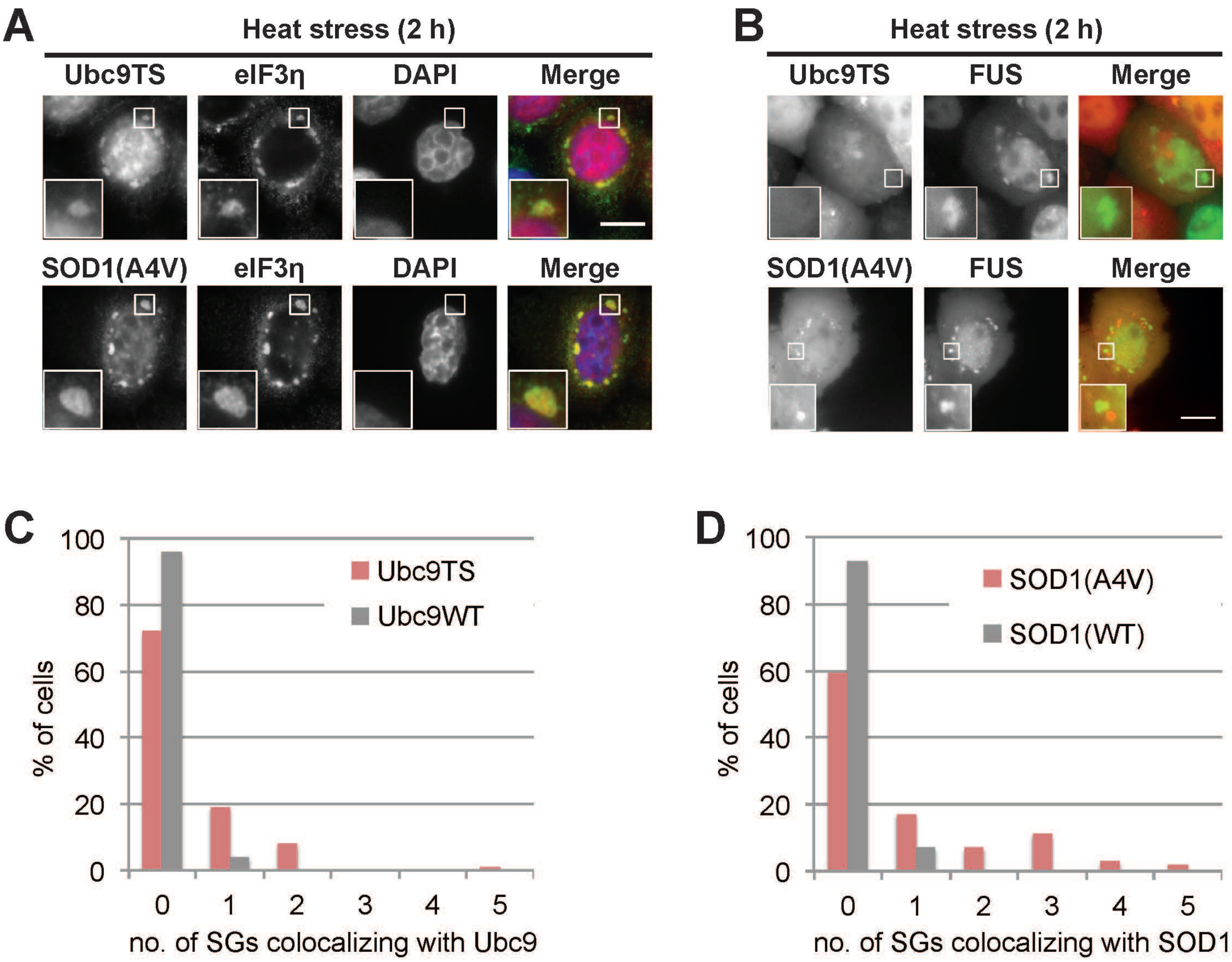

Appendix Figure S2. Colocalization of misfolded proteins and heat stress-induced SGs.

(A) HeLa cells expressing SOD1(A4V)-GFP or Ubc9TS-mCherry were incubated at 43°C for 2 hours, fixed and stained for eIF3η. Misfolded proteins localized to eIF3η-positive SGs. Scale bar = 10 μm.

(B) In some cells treated with heat stress (43°C for 2 hours), misfolded Ubc9TS or SOD1(A4V) localized to separate foci and not to SGs. Scale bar = 10 μm.

(C) Number of SGs colocalizing with Ubc9TS or Ubc9WT was calculated for 100 cells expressing Ubc9TS (381 SGs) and 100 cells expressing Ubc9WT (329 SGs), using the data shown in Fig 1D. Only cells with segmented SGs were analyzed. Colocalization is defined by fluorescent ratio > 1.4.

(D) Number of SGs colocalizing with SOD1(A4V) or SOD1(WT) was calculated for 100 cells expressing SOD1(A4V) (399 SGs) and 100 cells expressing SOD1(WT) (374 SGs), using the data shown in Fig 1G. Only cells with segmented SGs were analyzed. Colocalization is defined by fluorescent ratio > 1.4.

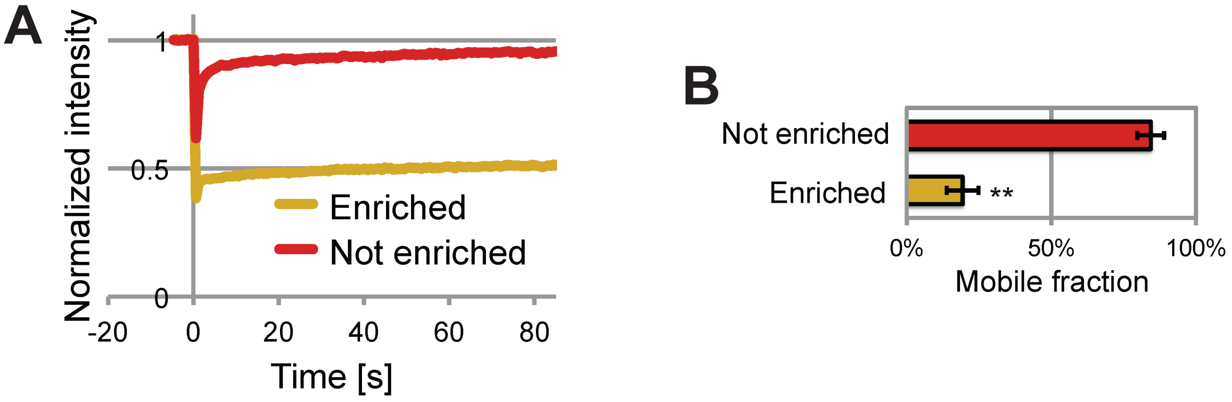

Appendix Figure S3. FRAP analysis of SOD1(A4V) in SGs.
(A) FRAP analysis of SOD1(A4V)-GFP in SGs in HeLa cells exposed to 2 hours of heat stress. Photobleaching was performed on SGs with high SOD1 enrichment ("enriched") or SGs with low SOD1 enrichment ("not enriched").
(B) Mobile fraction of SOD1(A4V) calculated from the FRAP analysis in C. Error bars = SEM. ** $p < 0.01$ (t-test).

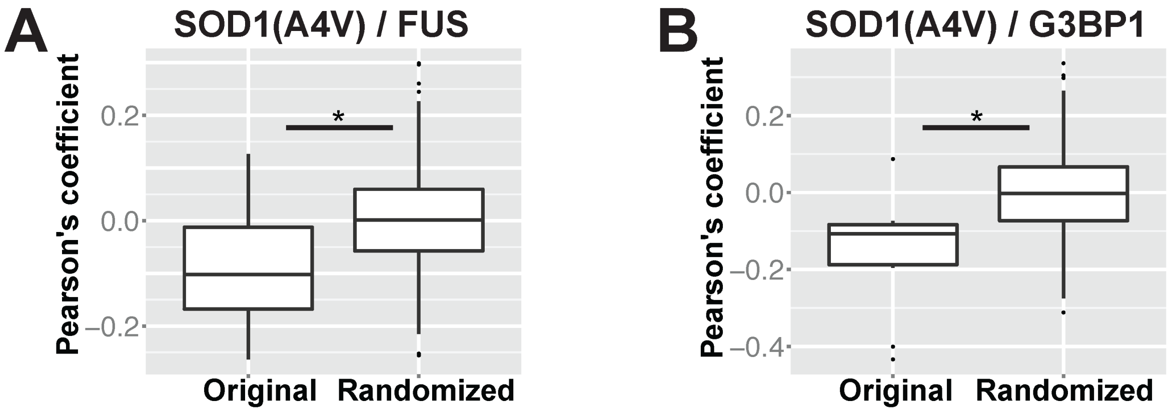

Appendix Figure S4. Colocalization analysis of SOD1(A4V) and RBPs inside SGs.
(A) Quantification of colocalization of SOD1(A4V)-GFP and FUS-mCherry in 10 SGs imaged by structured illumination microscopy, as shown in Figure 2H. Pearson's coefficient was quantified as a measure of colocalization within the SG. Negative values reflect anti-colocalization (tendency of signals to be excluded). T-test was used for comparison with control images created by Costes' randomization, * $p < 0.05$.
(B) Quantification of colocalization of SOD1(A4V)-GFP and G3BP1-mCherry in 10 SGs imaged by structured illumination microscopy, as shown in Figure 2I. T-test was used for comparison with images created by Costes' randomization, * $p < 0.05$.

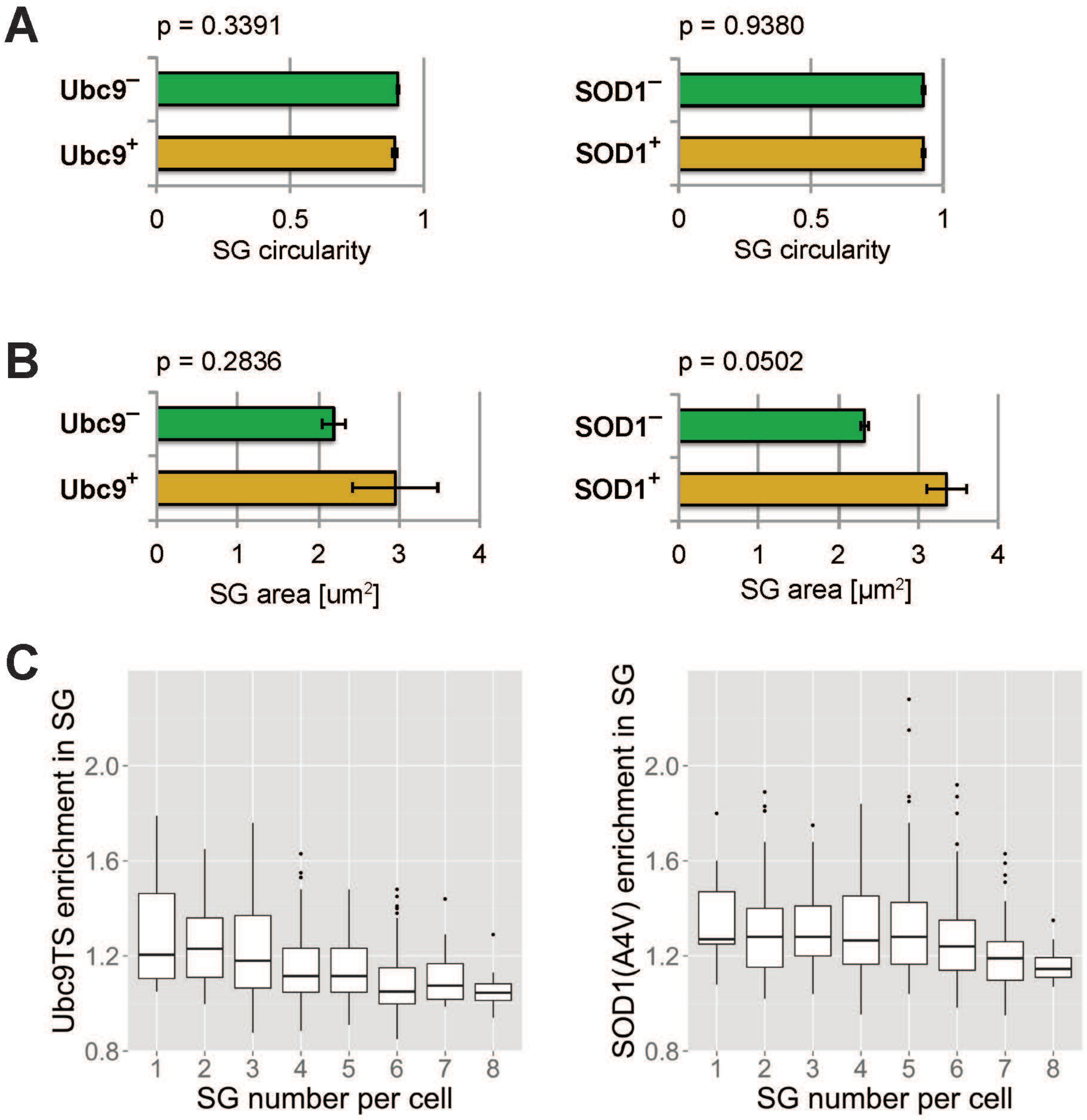

Appendix Figure S5. Properties of SGs enriched for Ubc9TS or SOD1(A4V).

(A) SG circularity was measured for SGs enriched for Ubc9TS (Ubc9+) and SGs not enriched for Ubc9TS (Ubc9-), using the data from Fig 1D and the fluorescent ratio 1.4 as a threshold. The same was measured for SGs enriched for SOD1(A4V) (SOD1+) and SGs not enriched for SOD1(A4V) (SOD1-), using the data from Fig 1G. P-values were calculated from the mean values of each sample (n=3; t-test). Error bars = SEM.

(B) SG area was measured for SGs enriched for Ubc9TS (Ubc9+) and SGs not enriched for Ubc9TS (Ubc9-), using the data from Fig 1D and the fluorescent ratio 1.4 as a threshold. The same was measured for SGs enriched for SOD1(A4V) (SOD1+) and SGs not enriched for SOD1(A4V) (SOD1-), using the data from 1G. P-values were calculated from the mean values of each sample (n=3; t-test). Error bars = SEM.

(C) Enrichment of Ubc9TS in SGs was calculated for all 381 SGs in 100 cells expressing Ubc9TS, using the data shown in Appendix Fig S2C. The SGs were divided based on number of SGs in the corresponding cell and the values were plotted in R. The same was measured for all 399 SGs in 100 cells expressing SOD1(A4V), using the data shown in Appendix Fig S2D.

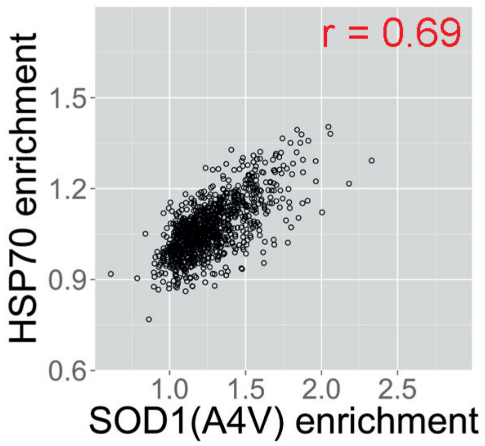

Appendix Figure S6. Correlation between HSP70 and SOD1(A4V) enrichment in SGs.
HeLa cells expressing FUS-mCherry and SOD1(A4V)-GFP were incubated at 43°C for 2 hours, fixed and stained with antibody against HSP70. Automated imaging assay was used to quantify the enrichment of HSP70 and SOD1(A4V) in SGs (1000 SGs plotted). Pearson's correlation coefficient (r) is shown.

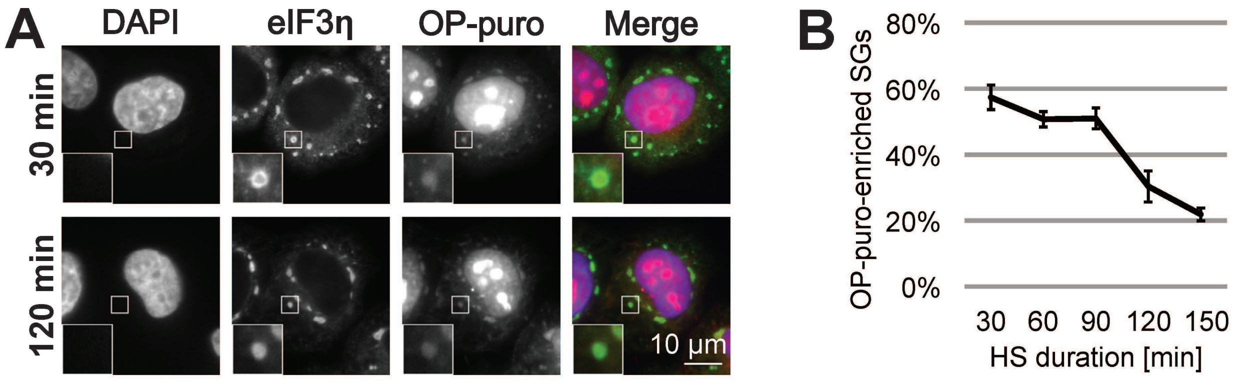

Appendix Figure S7. Accumulation of DRiPs in SGs.
(A) HeLa cells were incubated with 25 μM OP-puro for 30 or 120 minutes at 43°C. The cells were then fixed, followed by OP-puro labeling and immunofluorescence staining of eIF3η. OP-puro signal in SGs is visible. Additionally, a strong nuclear signal is observed, consistent with previous reports and possibly caused by nuclear translation (Seguin et al. 2014; Liu et al. 2012; David et al. 2012).
(B) Percentage of SGs enriched for OP-puro by duration of heat stress. HeLa cells were incubated with 25 μM OP-puro at 43°C for 30, 60, 90, 120 or 150 minutes. The cells were then fixed, followed by OP-puro labeling and immunofluorescence staining of eIF3η (used for SG segmentation). Automated imaging assay was used to quantify the percentage of SGs highly enriched for OP-puro at given time points (using threshold 1.5 for fluorescence ratio). Mean values from 3 independent experiments are shown, each sample (1 replicate of 1 time point) with > 5000 SGs (average 9652).

References

David, Alexandre, Brian P. Dolan, Heather D. Hickman, Jonathan J. Knowlton, Giovanna Clavarino, Philippe Pierre, Jack R. Bennink, and Jonathan W. Yewdell. 2012. "Nuclear Translation Visualized by Ribosome-Bound Nascent Chain Puromycylation." *The Journal of Cell Biology* 197 (1): 45–57.

Lakowicz, Joseph R. 2013. *Principles of Fluorescence Spectroscopy*. Springer Science & Business Media.

Liu, Jing, Yangqing Xu, Dan Stoleru, and Adrian Salic. 2012. "Imaging Protein Synthesis in Cells and Tissues with an Alkyne Analog of Puromycin." *Proceedings of the National Academy of Sciences of the United States of America* 109 (2): 413–18.

Seguin, S. J., F. F. Morelli, J. Vinet, D. Amore, S. De Biasi, A. Poletti, D. C. Rubinsztein, and S. Carra. 2014. "Inhibition of Autophagy, Lysosome and VCP Function Impairs Stress Granule Assembly." *Cell Death and Differentiation* 21 (12): 1838–51.

Slavík, J. 1982. "Anilinonaphthalene Sulfonate as a Probe of Membrane Composition and Function." *Biochimica et Biophysica Acta* 694 (1): 1–25.

Yan-Ling, Zhang, Pan Xian-Ming, and Zhou Jun-Mei. 1998. "Surface Hydrophobicity and Thermal Aggregation of Adenylate Kinase." *IUBMB Life* 44 (5). Informa Healthcare: 949–60.
