# Supplementary material for: An aberrant phase transition of stress granules triggered by misfolded protein and prevented by chaperone function
Source: EMBO J. 2017 Apr 4;36(12):1669–87. doi: 10.15252/embj.201695957 (PMC5470046; doi:10.15252/embj.201695957)
Supplement: Supplementary file 2 — Expanded View Figures PDF [file EMBJ-36-1669-s002.pdf]

Expanded View Figures

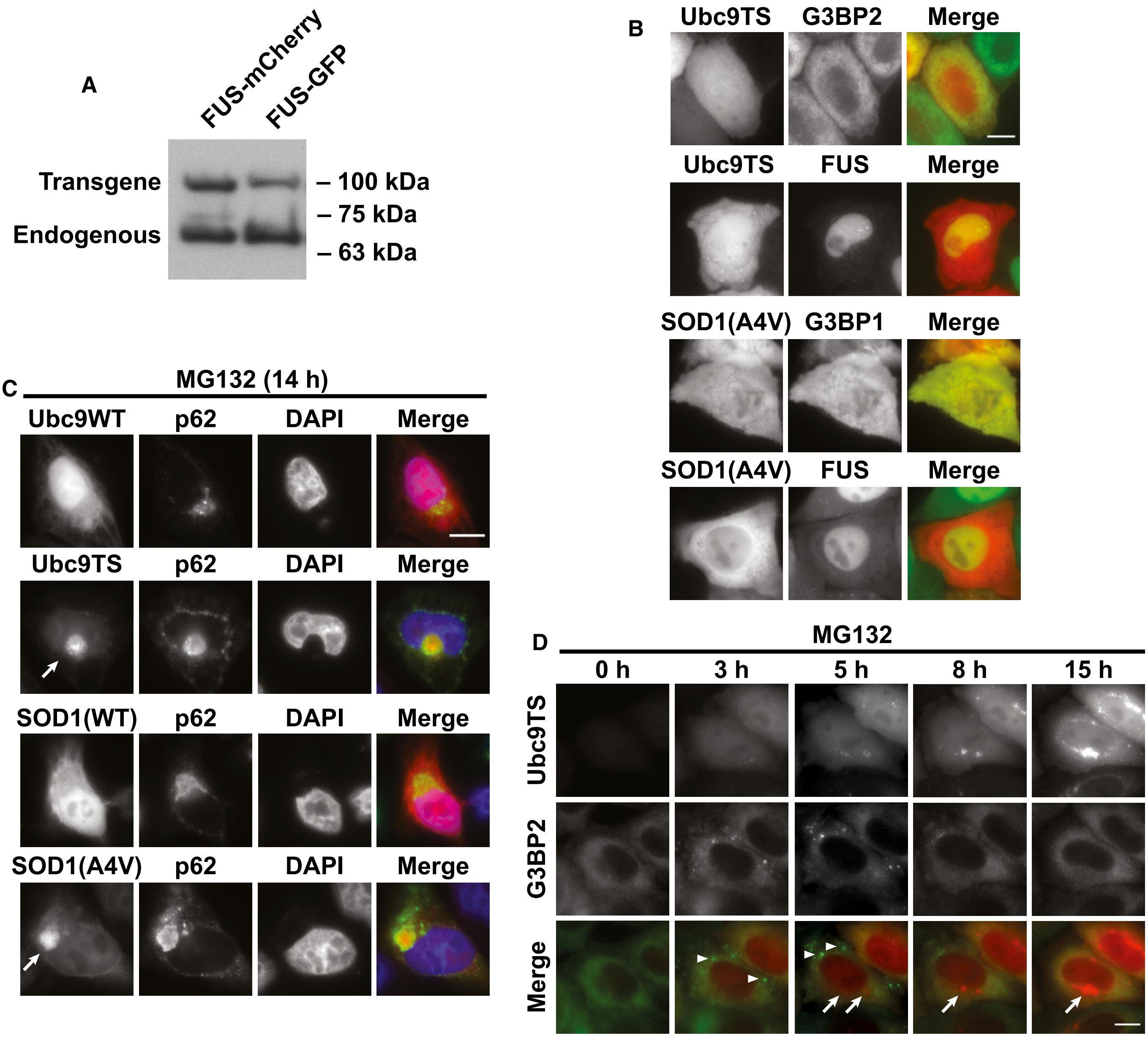

Figure EV1. Characterization of HeLa cells expressing SG markers and mutant misfolding-prone proteins.

A HeLa stable cell lines expressing BAC-encoded FUS-mCherry or FUS-GFP were analyzed by Western blotting using anti-FUS antibody. Positions of endogenous FUS and the transgenes are indicated.

B HeLa cells expressing BAC-encoded SG markers (FUS-mCherry, G3BP1-mCherry, FUS-GFP, or G3BP2-GFP) were transfected with plasmids expressing SOD1(A4V)-GFP or Ubc9TS-mCherry. Live cells were imaged under normal conditions (37°C, no treatment). FUS is localized in the nucleus, and G3BP is diffusely distributed in the cytoplasm. Misfolding-prone proteins Ubc9TS and SOD1(A4V) are also diffusely distributed. Scale bar = 10 μm.

C HeLa cells expressing SOD1(A4V)-GFP, SOD1(WT)-GFP, Ubc9TS-mCherry, or Ubc9WT-mCherry were treated with MG132 (10 μM) for 14 h. Cells were fixed and stained for p62. p62 localized to large perinuclear inclusions (arrows) together with misfolded proteins but not WT variants of SOD1 or Ubc9. Scale bar = 10 μm.

D HeLa cells expressing G3BP2-GFP and Ubc9TS-mCherry were imaged during the treatment with MG132 (10 μM). G3BP2 assembled into SGs (arrowheads), Ubc9TS accumulated over time and localized to aggresome (arrows). Scale bar = 10 μm. Images are from Movie EV1.

 © 2017 The Authors

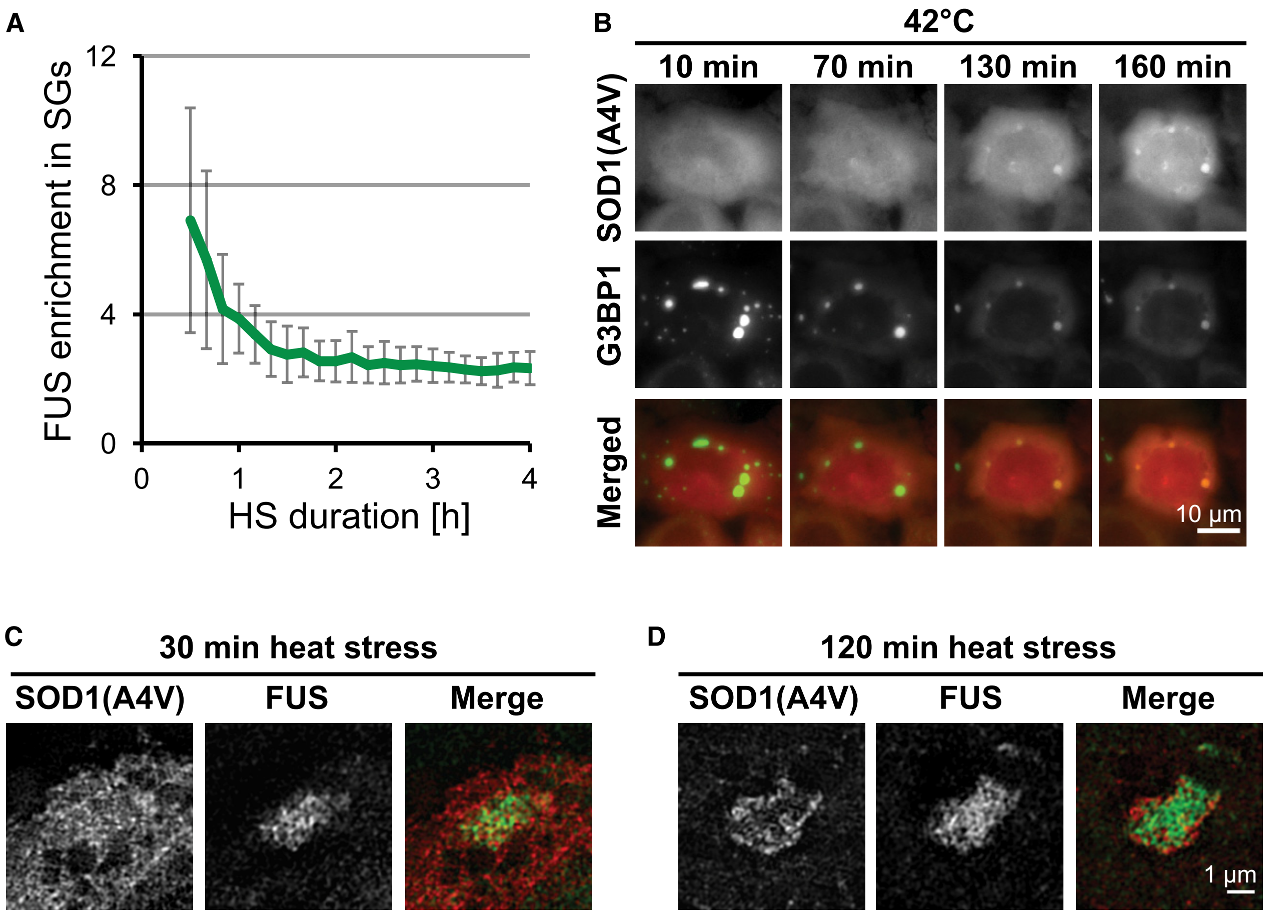

Figure EV2. Misfolded proteins accumulate in SGs and form immobile domains with prolonged heat stress.

A Quantification of relative FUS enrichment (intensity in SG/intensity outside SG) in the SGs from Fig 2A at different time points of heat stress. Mean values are shown (13–27 SGs in each frame). Error bars = standard deviation.

B HeLa cells expressing G3BP1-mCherry and SOD1(A4V)-GFP were imaged at 42°C. SG formation was followed by accumulation of SOD1 in SGs. G3BP1 was partially depleted from SGs with prolonged stress.

C Super-resolution image of a SG after 30 min of heat stress. Cells are expressing FUS-mCherry and SOD1(A4V)-GFP.

D Super-resolution image of a SG after 2 h of heat stress. Cells are expressing FUS-mCherry and SOD1(A4V)-GFP.

© 2017 The Authors

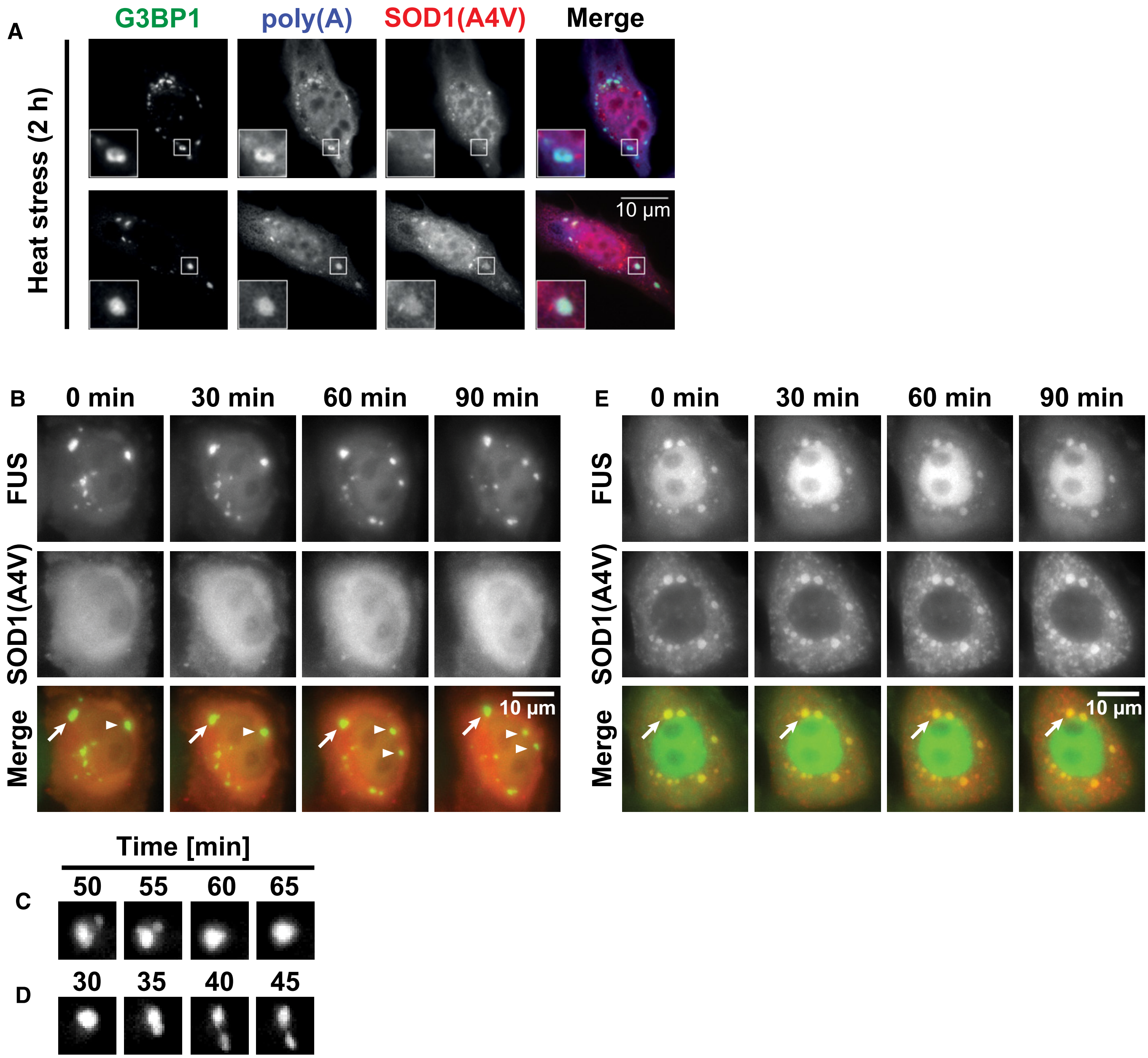

Figure EV3. Properties of SOD1-positive and SOD1-negative SGs.

A HeLa cells expressing SOD1(A4V)-GFP were heat-stressed for 2 h and fixed. G3BP1 was then detected by immunofluorescence, and poly(A) RNA was detected by fluorescence *in situ* hybridization. In the same sample, some SGs were clearly enriched for SOD1(A4V) (lower cell), while other SGs were not (upper cell). Both types of SGs contained poly(A) mRNA signal.

B HeLa cells expressing FUS-mCherry and SOD1(A4V)-GFP were heat-stressed for 2 h and then imaged at 37°C (time indicates duration of recovery). SOD1-negative SGs showed fusion (arrows) and fission (arrowheads).

C Fusion of SGs from the cell shown in (B).

D Fission of a SG from the cell shown in (B).

E In other cells treated the same way as in (B), SOD1-positive SGs (arrows) showed less dynamic behavior.

 © 2017 The Authors

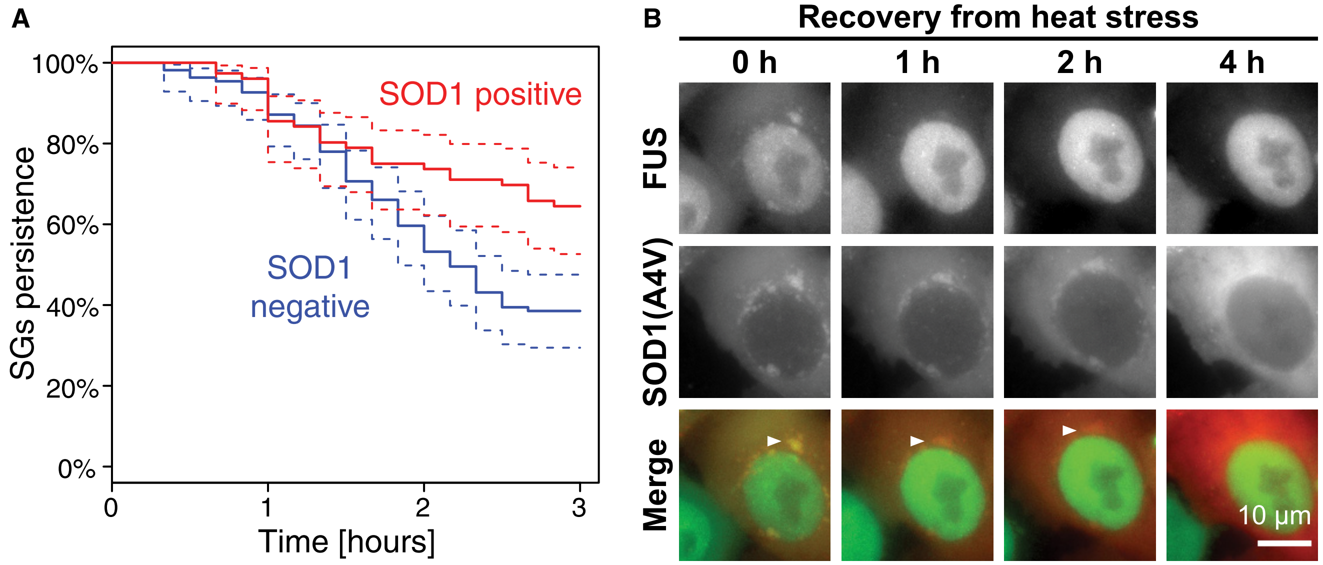

Figure EV4. Disassembly of aberrant SGs.

A HeLa cells expressing FUS-mCherry and SOD1(A4V)-GFP were heat-stressed for 2 h and then imaged during recovery at 37°C. Six independent experiments were performed. Complete SG disassembly was scored in cells containing SOD1-negative SGs (109 cells) or cells containing SOD1-positive SGs (76 cells) and plotted using survival analysis in R. Time indicates duration of recovery. Dashed lines = 95% confidence intervals. $P < 0.01$ (log-rank test).

B HeLa cells expressing FUS-mCherry and SOD1(A4V)-GFP were treated with heat stress for 2 h and then imaged during recovery at 37°C to observe the disassembly of SOD1-containing SGs (arrowheads).

© 2017 The Authors

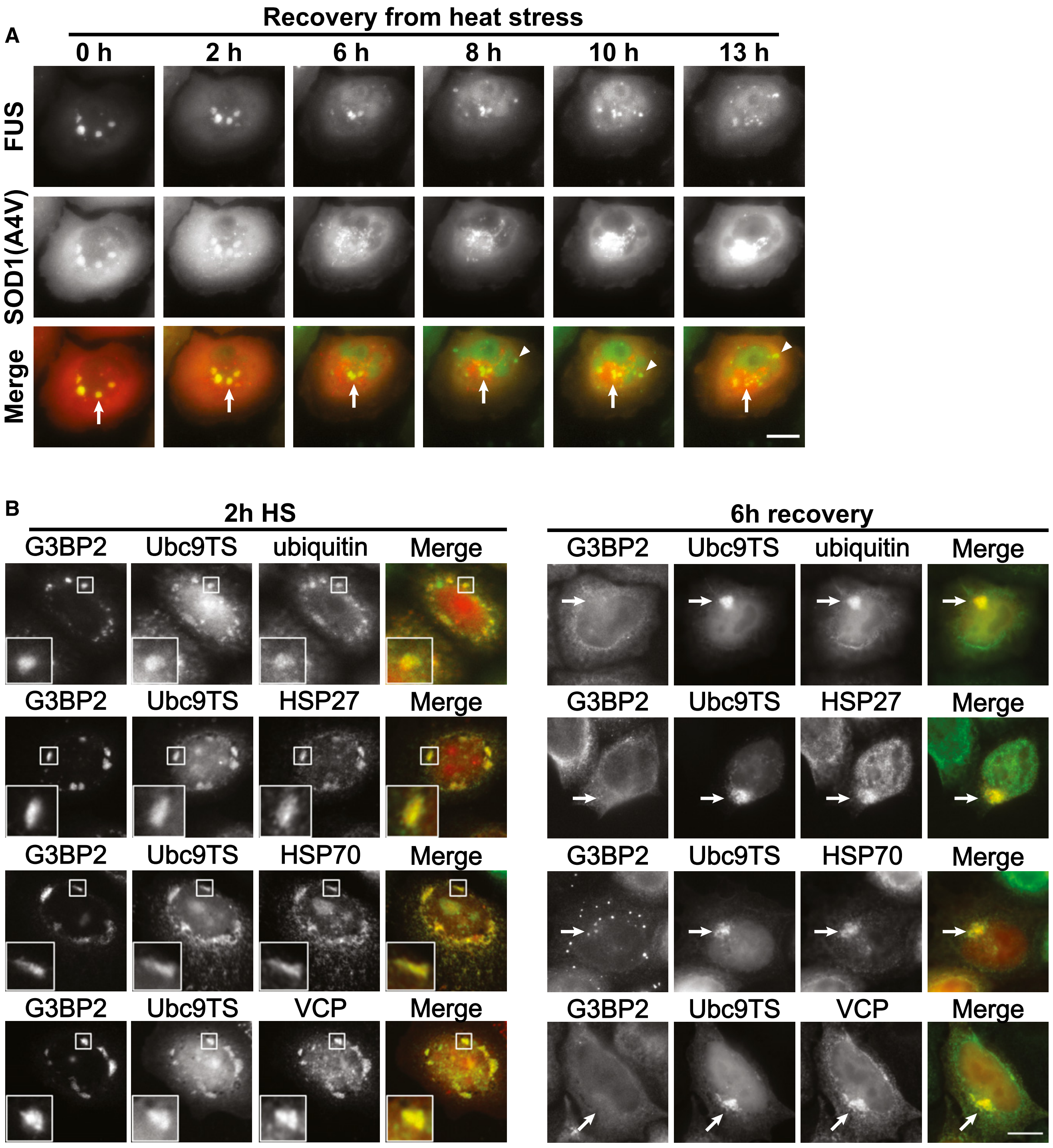

Figure EV5. Transport of SG components to the aggresome.

A HeLa cells expressing FUS-mCherry and SOD1(A4V)-GFP were treated with heat stress for 2 h and then imaged during recovery at 37°C. SOD1-containing SGs (arrows) are transported toward the aggresome, where they slowly disappear. Meanwhile, SOD1 accumulates in the aggresome and new SGs devoid of SOD1 are formed (arrowheads). Scale bar = 10 μm. Brightness was normalized across frames.

B HeLa cells expressing G3BP2-GFP and Ubc9TS-mCherry were treated either with 2-h heat stress (left) or 2-h heat stress followed by 6 h of recovery at 37°C (right). The cells were then fixed and stained for poly-ubiquitin, HSP27, HSP70, or VCP. After 2 h of heat stress, components of PQC machinery colocalized with Ubc9TS in SGs (insets). After additional 6 h of recovery, the proteins colocalized with Ubc9TS in the aggresome (arrows). Merge = immunofluorescence (green) + Ubc9TS (red). Scale bar = 10 μm.

 © 2017 The Authors
