# Supplementary material for: An aberrant phase transition of stress granules triggered by misfolded protein and prevented by chaperone function
Source: EMBO J. 2017 Apr 4;36(12):1669–87. doi: 10.15252/embj.201695957 (PMC5470046; doi:10.15252/embj.201695957)
Supplement: Supplementary file 3 — Movie EV1 [file EMBJ-36-1669-s003.zip › MovieEV1/MovieEV1.rtf]

Movie EV1. Formation of SGs and aggresome induced by proteasome inhibition. HeLa cells expressing G3BP2-GFP (green) and Ubc9TS-mCherry (red) were treated with 10 µM MG132 and immediately imaged with 5 minute intervals. No colocalization between SGs and aggresome was observed.
