# Supplementary material for: An aberrant phase transition of stress granules triggered by misfolded protein and prevented by chaperone function
Source: EMBO J. 2017 Apr 4;36(12):1669–87. doi: 10.15252/embj.201695957 (PMC5470046; doi:10.15252/embj.201695957)
Supplement: Supplementary file 4 — Movie EV2 [file EMBJ-36-1669-s004.zip › MovieEV2/MovieEV2.rtf]

Movie EV2. 3D visualization of SGs imaged by structured illumination microscopy.Montage showing 360° rotation of a SG containing FUS-mCherry and SOD1(A4V)-GFP (left, also shown in Figure 2H) and a SG containing G3BP1-mCherry and SOD1(A4V)-GFP (right, also shown in Figure 2I). SGs were induced with 2 h heat stress.
