# Supplementary material for: An aberrant phase transition of stress granules triggered by misfolded protein and prevented by chaperone function
Source: EMBO J. 2017 Apr 4;36(12):1669–87. doi: 10.15252/embj.201695957 (PMC5470046; doi:10.15252/embj.201695957)
Supplement: Supplementary file 5 — Movie EV3 [file EMBJ-36-1669-s005.zip › MovieEV3/MovieEV3.rtf]

Movie EV3. Two populations of SGs in one cell. HeLa cells expressing FUS-mCherry (green) and SOD1(A4V)-GFP (red) were heat-stressed for 2 hours and subsequently imaged with 5 minute intervals. SOD1-positive SGs (yellow) are static and localized close to nucleus, SOD1-negative SGs (green) are dynamic and undergoing fusion events.
