# Supplementary material for: An aberrant phase transition of stress granules triggered by misfolded protein and prevented by chaperone function
Source: EMBO J. 2017 Apr 4;36(12):1669–87. doi: 10.15252/embj.201695957 (PMC5470046; doi:10.15252/embj.201695957)
Supplement: Supplementary file 6 — Movie EV4 [file EMBJ-36-1669-s006.zip › MovieEV4/MovieEV4.rtf]

Movie EV4. Transport of persistent SGs to the aggresome. HeLa cells expressing G3BP2-GFP (green) and Ubc9TS-mCherry (red) were heat-stressed for 2 hours and subsequently imaged with 5 minute intervals. Ubc9TS-positive SGs (yellow) are slowly transported towards a perinuclear inclusion containing large amount of Ubc9TS. Meanwhile, new Ubc9TS-negative SGs are formed (green).
