# Supplementary material for: An aberrant phase transition of stress granules triggered by misfolded protein and prevented by chaperone function
Source: EMBO J. 2017 Apr 4;36(12):1669–87. doi: 10.15252/embj.201695957 (PMC5470046; doi:10.15252/embj.201695957)
Supplement: Supplementary file 7 — Movie EV5 [file EMBJ-36-1669-s007.zip › MovieEV5/MovieEV5.rtf]

Movie EV5. Accumulation of misfolded proteins in SG during heat stress.A HeLa cell expressing G3BP2-GFP (green) and the misfolding-prone protein VHL-mCherry (Kaganovich et al., 2008) was imaged at 42°C with 150 second intervals (starting 20 min after exposure to heat stress). Initially, SGs are devoid of VHL. With prolonged heat stress (1 hour onwards), the SG in the center starts to recruit misfolded VHL (color change from green to yellow), loses its dynamic behavior and remains close to the nucleus. Meanwhile, new SGs are formed and exhibit dynamic behavior such as fusion, fission and movement (green). Brightness was normalized across frames.
